# The anti-aging factor Klotho protects against acquired long QT syndrome induced by uremia and promoted by fibroblast growth factor 23

**DOI:** 10.1186/s12916-021-02209-9

**Published:** 2022-01-19

**Authors:** José Alberto Navarro-García, Rafael Salguero-Bodes, Laura González-Lafuente, Laura Martín-Nunes, Elena Rodríguez-Sánchez, Teresa Bada-Bosch, Eduardo Hernández, Evangelina Mérida-Herrero, Manuel Praga, Jorge Solís, Fernando Arribas, Héctor Bueno, Makoto Kuro-O, María Fernández-Velasco, Luis Miguel Ruilope, Carmen Delgado, Gema Ruiz-Hurtado

**Affiliations:** 1 Cardiorenal Translational Laboratory, Institute of Research imas12, Hospital Universitario 12 de Octubre, Avenida de Córdoba s/n, 28041 Madrid, Spain; 2 Cardiology Department, Hospital Universitario 12 de Octubre, Madrid, Spain; 3 Facultad de Medicina, Universidad Complutense de Madrid, Madrid, Spain; 4 CIBER-CV, Hospital Universitario 12 de Octubre, Madrid, Spain; 5 Biomedical Research Institute Alberto Sols (CSIC-UAM)/CIBER-CV, Arturo Duperier 4, 28029 Madrid, Spain; 6 Service of Nephrology, Hospital Universitario 12 de Octubre, Madrid, Spain; 7 Centro Nacional de Investigaciones Cardiovasculares (CNIC), Madrid, Spain; 8 Division of Anti-aging Medicine, Centre for Molecular Medicine, Jichi Medical University, Shimotsuke, Tochigi, Japan; 9 IdiPAZ Institute for Health Research/Centro de Investigación Biomédica en Red de Enfermedades Cardiovasculares, CIBER-CV, Madrid, Spain; 10 European University of Madrid, Madrid, Spain

**Keywords:** CKD, Dialysis, FGF23, Klotho, Long QT, Potassium channels

## Abstract

**Background:**

Chronic kidney disease (CKD) is associated with increased propensity for arrhythmias. In this context, ventricular repolarization alterations have been shown to predispose to fatal arrhythmias and sudden cardiac death. Between mineral bone disturbances in CKD patients, increased fibroblast growth factor (FGF) 23 and decreased Klotho are emerging as important effectors of cardiovascular disease. However, the relationship between imbalanced FGF23-Klotho axis and the development of cardiac arrhythmias in CKD remains unknown.

**Methods:**

We carried out a translational approach to study the relationship between the FGF23–Klotho signaling axis and acquired long QT syndrome in CKD-associated uremia. FGF23 levels and cardiac repolarization dynamics were analyzed in patients with dialysis-dependent CKD and in uremic mouse models of 5/6 nephrectomy (Nfx) and Klotho deficiency (hypomorphism), which show very high systemic FGF23 levels.

**Results:**

Patients in the top quartile of FGF23 levels had a higher occurrence of very long QT intervals (> 490 ms) than peers in the lowest quartile. Experimentally, FGF23 induced QT prolongation in healthy mice. Similarly, alterations in cardiac repolarization and QT prolongation were observed in Nfx mice and in Klotho hypomorphic mice. QT prolongation in Nfx mice was explained by a significant decrease in the fast transient outward potassium (K^+^) current (*I*_*tof*_), caused by the downregulation of K^+^ channel 4.2 subunit (*Kv4.2*) expression. *Kv4.2* expression was also significantly reduced in ventricular cardiomyocytes exposed to FGF23. Enhancing Klotho availability prevented both long QT prolongation and reduced *I*_*tof*_ current. Likewise, administration of recombinant Klotho blocked the downregulation of *Kv4.2* expression in Nfx mice and in FGF23-exposed cardiomyocytes.

**Conclusion:**

The FGF23–Klotho axis emerges as a new therapeutic target to prevent acquired long QT syndrome in uremia by minimizing the predisposition to potentially fatal ventricular arrhythmias and sudden cardiac death in patients with CKD.

**Graphical abstract:**

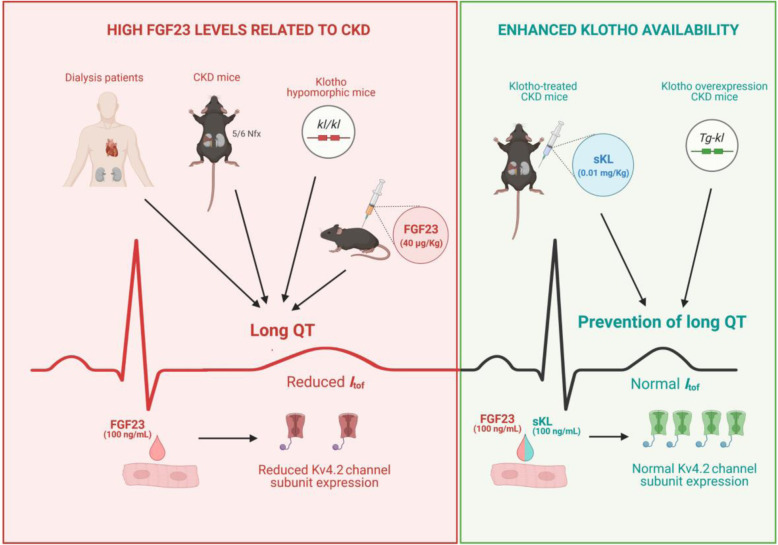

**Supplementary Information:**

The online version contains supplementary material available at 10.1186/s12916-021-02209-9.

## Background

The risk of cardiovascular disease and mortality increases as renal function declines [1, 2], with the highest risk in patients with dialysis-dependent chronic kidney disease (CKD) [3]. In addition, patients with dialysis-dependent CKD frequently develop mineral bone disorders (MBDs), which aggravate the decline in kidney function [4]. Among the components of bone mineral metabolism, fibroblast growth factor (FGF) 23 appears to be an important contributor to the elevated cardiovascular risk in patients with CKD [5, 6], especially for those receiving dialysis [7]. Dialysis patients have high serum levels of FGF23 [8]. Nevertheless, the causal role of FGF23 on cardiovascular risk remains unclear [9]. FGF23 is a bone-derived hormone that is stimulated in response to high serum phosphate levels and binds to FGF receptors (FGFRs) in the kidney to trigger phosphate excretion. FGF23 binding to FGFR requires the presence of the cofactor Klotho [10], a powerful regulator of aging and life-span [11]. Klotho is a transmembrane protein expressed mainly in kidney [11], although a soluble form is released into circulation by the action of secretases [12]. Soluble Klotho has several pleiotropic functions that are poorly understood, including an apparently cardioprotective action [13]. Reduced Klotho expression in CKD leads to resistance to the phosphaturic effect of FGF23 [14], increasing serum phosphate levels that pathologically stimulate FGF23 synthesis. Accordingly, CKD-related MBDs drive a premature aging phenotype in patients with CKD as consequence of the increase in pro-aging factors such as phosphates and FGF23 and the decrease in anti-aging factors including Klotho [15, 16]. Indeed, CKD promotes the greatest discrepancy between chronological and biological age, and it has been reported that cardiovascular mortality in 20-year-old dialysis patients is similar to that of healthy 80-year-olds [17]. As aging is an independent cardiovascular disease risk factor [18], CKD-related premature aging may additionally increase cardiovascular risk in patients with this pathology.

Renal dysfunction is linked to high cardiovascular risk, morbidity and mortality, at least in part, due to the high prevalence of heart failure (HF) and arrhythmias observed in patients with CKD [3, 19]. Indeed, cardiovascular deaths account for about 50% of all deaths in CKD, which are principally due to fatal ventricular arrhythmias [20]. The QT interval represents the electrical activity corresponding to the duration of the ventricular depolarization and repolarization phases of electrocardiogram (ECG). Prolonged QT intervals increase the risk of the polymorphic ventricular tachycardia as *Torsades de Pointes*, sudden cardiac death (SCD), and all-cause mortality in the general population [21]. It is well established that the prevalence of prolonged QT interval increases as renal function deteriorates [22]. Dialysis patients frequently develop long QT syndrome [23, 24]. In patients on dialysis, prolonged rate-corrected QT (QTc) duration has been associated with all-cause [25] and cardiovascular mortality independently of other risk factors [23]. Many factors can influence cardiac repolarization in CKD including hypertension, diabetes mellitus, HF, electrolyte disorders, and drug abuse [26–29]. However, the influence of the mineral metabolism component FGF23 on cardiac repolarization and the putative cardioprotective role of Klotho are still unknown.

In the present study, we characterized the underlying mechanisms and factors involved directly in prolonged cardiac repolarization in clinical and experimental uremia, and we tested the hypothesis that improving the bioavailabity of the antiaging factor Klotho would prevent the electrical repolarization disturbances related to uremia.

## Methods

### Clinical study in patients with dialysis-dependent CKD

The study population included a total of 33 patients (17 men and 16 women) with dialysis-dependent CKD, corresponding to the complete dialysis cohort of patients receiving ambulatory dialysis in the Dialysis Service of *Hospital Universitario 12 de Octubre*. Inclusion criteria were age ≥ 18 years and being on the chronic dialysis treatment while the presence of clinical diagnosis of atrial fibrillation was an exclusion criteria. Patients were 19–87 years of age. The causes of renal failure were: chronic glomerulonephritis (24.5%), diabetic nephropathy (18.9%), interstitial nephritis (11.3%), nephroangiosclerosis (11.3%), polycystic kidney disease (5.7%), ischemic nephropathy (5.7%), malignant hypertension (5.7%), atypical hemolytic uraemic syndrome (3.8%), and others (13.1%). Blood samples were collected in EDTA tubes before starting dialysis therapy. Clinical biochemical parameters including cholesterol, calcium, phosphorus, 25-hydroxyvitamin D, and parathyroid hormone were measured by the Biochemistry Service of *Hospital Universitario 12 de Octubre*. Serum levels of C-terminal human FGF23 were measured by ELISA (Immunotopics Inc.). Patients were grouped into four quartiles according to the systemic levels of FGF23. ECGs were recorded using a 12-lead Holter Monitoring Device (CardioScan Premier 12, DM Software). ECGs were recorded for 48 h, from 1 h before starting dialysis therapy to the beginning of the next dialysis session. The recordings were analyzed and interpreted using specific software tools under the supervision of two cardiologists specialized in arrhythmology from the Cardiology Department of *Hospital Universitario 12 de Octubre*. QTc calculations were performed for every single beat during 48 h Holter recordings, and the variable of QTc for every particular patient was showed as a Gaussian distribution of measurements. Automatic beat-to-beat QTc measurements from all patients were classified according to the duration of the interval as not prolonged (≤ 450 ms), mildly prolonged (450–490 ms), and prolonged (> 490 ms) for analysis [30, 31]. The cut-off for long QT interval of 450 ms was used as previously described [32] and independently of the sex as it has been described that in population older than 40 years gender differences for QT interval become smaller [33]. The TpTe interval was measured manually following the “tangent” method in lead V_5_. This method measures the TpTe interval as the distance between the maximum positive or negative deflection of the T-wave from the isoelectric line and the intersection of the tangent to the downslope of the T-wave with the isoelectric line [34]. All measurements were carried out blinded to other patient data.

### Animal study

Animals were maintained at controlled temperature (23–25 °C) on a 12-h light/dark cycle with ad libitum access to water and a standard diet (ROD14, Altromin Spezialfutter GmbH & Co. KG). Animals were housed in groups of 4 per cage of 553 cm^2^ by 20.8 cm depth (polysulfone cage type II L, SODISPAN) with standard wood chip (ECO-PURE 7 Chips, Tapvei®).

Adult 14-week-old male C57BL/6 J mice (Charles River Laboratories International Inc.) were used to study the effect of FGF23 on heart rhythm by a single-dose intraperitoneal injection of recombinant mouse FGF23 (40 μg/kg) (R&D Systems) during ECG recording.

#### Experimental CKD model by 5/6 nephrectomy

Adult six-week-old male C57BL/6 J mice (Charles River Laboratories International Inc.) underwent sham or 5/6 nephrectomy (Nfx) surgery to induce CKD. Nfx was performed in a two-step surgery under isoflurane anesthesia (1.5% v/v, isoflurane/oxygen) as described [35]. After each surgery, a single dose subcutaneous injection of meloxicam (0.06 mL/kg) was used as analgesia. Sham and Nfx mice were randomly divided into two groups and treated daily with an intraperitoneal injection of vehicle solution (0.9% sodium chloride) or recombinant mouse Klotho (rKL, 0.01 mg/kg/day) [36] immediately after the second surgery and during 6 weeks. Mice were sacrificed six weeks after the second surgery. Similarly, 6-week-old male transgenic overexpressing Klotho mice (*Tg-Kl*) mice underwent Sham or Nfx surgery as described and no additional treatment was used.

#### Macroscopic parameters and serum biochemistry

Heart weight (HW) to body weight (BW) ratio was calculated as an index of cardiac hypertrophy. Cardiomyocyte surface area was measured as the total cell area using images obtained from confocal microscopy (MetaZeiss LSM 510, objective w.i. 40×, n.a. 1.2). Blood samples were collected in EDTA tubes just prior to sacrifice. Blood samples were centrifuged at 2500 rpm for 10 min at 4 °C for plasma collection. Plasma levels of urea and blood urea nitrogen (BUN) (BioAssays System), phosphorus (Abcam), and FGF23 (Immunotopics Inc.) were measured by ELISA.

#### Electrocardiogram

ECGs registries were made using a Small Animal Physiological Monitoring System (Harvard Apparatus). ECGs were recorded from mice lightly anesthetized with isoflurane (1.5% v/v, isoflurane/oxygen) the day before the sacrifice. Mice were located in prone position on the pre-heated pad at 37 °C for the registry, placed on the metal pads of ECG sensors. Paws were covered with conductive gel to guarantee an optimal electrical connection. A cross-platform Java program was used to convert ECG registries into LabChart binary. Converted files were analyzed using LabChart 7.0 software (ADInstruments). QT, JT, and TpTe interval measurements were obtained from LabChart analysis. QT interval duration was corrected using the *Mitchell* formula [37]:

$$ QTc=\frac{QT}{\sqrt{\frac{RR}{100}}} $$

where QTc is the corrected QT interval, QT is the interval between Q wave and T wave end, and RR is the interval between one R wave and the R wave of the next QRS complex.

#### Adult mouse ventricular myocyte isolation

Mice were heparinized and anaesthetized with ketamine (100 mg/kg) and xylazine (10 mg/kg) *via* intraperitoneal injection. Hearts from anesthetized mice were rapidly removed and cannulated via the ascending aorta onto a Langendorff perfusion system. Hearts were retrograde perfused for 3 min with calcium-free Tyrode’s solution supplemented with EGTA (0.2 mmol/L) and followed by Tyrode’s solution containing type II collagenase (1 mg/mL, Worthington), CaCl_2_ (0.1 mmol/L), and BSA (1 mg/mL) for 3 to 5 min. Hearts were removed from the apparatus and the ventricles were cut into small pieces to facilitate digestion. Digested hearts were filtered through a nylon mesh strainer (250 μm) to remove pieces of tissue and finally centrifuged at room temperature at 300 rpm for 3 min. The supernatant was removed and the pellet was resuspended in Tyrode’s solution with CaCl_2_ (0.5 mmol/L) and BSA (2 mg/mL) and centrifuged again. The resulting pellet was resuspended in Tyrode’s solution with CaCl_2_ (1 mmol/L) and BSA (2 mg/mL). Tyrode’s solution composition was (in nM) as follows: 130 NaCl, 5.4 KCl, 0.4 NaH_2_PO_4_, 0.5 MgCl_2_, 35 HEPES, and 22 glucose, and pH was adjusted to 7.4 using LiOH [38].

#### Neonatal mouse ventricular myocyte isolation

Cells were isolated from a total of 12 mice using the Pierce Primary Cardiomyocyte Isolation Kit (ThermoScientific). Isolated neonatal cardiomyocytes were maintained in culture for 5 days. On day 5, neonatal cardiomyocytes where preincubated for 3 h with vehicle or rKL (100 ng/mL) and then incubated with vehicle or FGF23 (100 ng/mL) for 48 h. Neonatal cardiomyocytes were then harvested and centrifuged at 1000 rpm for 3 min. Supernatants were discarded and pellets were stored at − 80 °C until use.

#### Electrophysiological studies

Isolated ventricular cardiomyocytes maintained in Tyrode’s solution with 1 mmol/L CaCl_2_ were used in electrophysiological studies within 4 h of isolation. The whole-cell voltage-clamp method was used. The fast transient outward current (*I*_*tof*_) was recorded using an Axopatch 200B amplifier (Molecular Devices). The patch pipette resistance was 1.0–2 MΩ. Patch clamp experiments were carried out at room temperature. Current traces were digitized with Digidata 1440A and analyzed using pClamp10 software (Molecular Devices).

Current density was obtained from the normalization of the current amplitude to the membrane capacitance (Cm). Cm was obtained by applying ±10 mV voltage steps from − 60 mV. The following equation was used to calculate Cm:

$$ Cm=\frac{\uptau_{\mathrm{c}}\times {I}_0}{\Delta  {V}_m\times \left[1-\frac{I_{\infty }}{I_0}\right]} $$

where *τ*_*c*_ is the time constant of the membrane capacitance, *I*_0_ is the maximum capacitance current value, ∆*V*_m_ is the amplitude of the voltage step, and *I*_∞_ is the amplitude of the steady-state current.

*I*_*tof*_ was obtained as described [39]. Total *I*_*to*_ was obtained by the application of 300 ms depolarizing voltage pulses from the holding potential of − 80 mV, from − 50 mV to + 50 mV at a frequency of 0.2 Hz. *I*_*tof*_ was inactivated by applying a short prepulse of 100 ms step at − 30 mV from a holding potential of − 80 mV, followed by depolarizing pulses from − 50 mV to + 50 mV. *I*_*tof*_ was calculated as the difference between the current recording with and without inactivating prepulse. The extracellular solution contained (in mM) the following: 135 NaCl, 10 glucose, 10 HEPES, 1 MgCl_2_, 1 CaCl_2_, 5.4 KCl, and 2 CoCl_2_; the pH was adjusted to 7.4 with NaOH. The pipette solution contained (in mM) the following: 135 KCl, 4 MgCl_2_, 5 EGTA, 10 HEPES, 10 glucose, 5 Na_2_ATP, and 5 disodium creatine phosphate 20; the pH was adjusted to 7.2 with KOH.

#### RNA isolation and quantitative real-time PCR

Total RNA from adult and neonatal cardiomyocytes was isolated using TiaZol (Qiagen). Quality and quantity of isolated RNA were assessed with on the NanoDrop One Microvolume UV-Vis Spectrophotometer (ThermoFisher Scientific). Reverse-transcription using High-Capacity cDNA Reverse Transcription Kit (Applied Biosystems) was used to obtain cDNA from 2 μg of RNA. cDNA samples were used for quantitative real-time PCR (qRT-PCR) using the FastStart Essential DNA Green Master (Roche) in 10 μL of total reaction volume on a LightCycler® 480 II (Roche) at optimized thermocycling settings. Ribosomal housekeeping gene 36b4 (*RPLP0*) was used to normalize relative gene expression, which was evaluated using the 2^−ΔΔCt^ method.

Primer sequences (5′-3′) used were as follows: m-Kcnd2-F: GCCGCAGCACCTAGTCGTT; m-Kcnd2-R: CACCACGTCGATGATACTCATGA; m-Rplp0-F: AGATGCAGCAGATCCGCAT; m-Rplp0-R: GTTCTTGCCCATCAGCACC.

### Statistics

Experimental data are reported as mean ± standard error of mean (SEM) and human data are presented as mean ± standard deviation (SD). Statistical significance was estimated using unpaired Student’s test, *χ*^2^ test, or ANOVA with Newman-Keuls multiple comparison test when appropriate. Gaussian distribution of the values was determined using Kolmogorov-Smirnov and Shapiro-Wilk tests. All *P*-values are two-tailed and *P*-values < 0.05 were considered statistically significant. All analyses were performed using GraphPad Prism v6.0 (GraphPad Software Inc.), Origin Pro v9.0 (OriginLab Corp.), or SPSS22 (IBM).

## Results

### Higher circulating FGF23 levels are associated with longer QT interval in patients with dialysis-dependent CKD

**Fig. 1:**
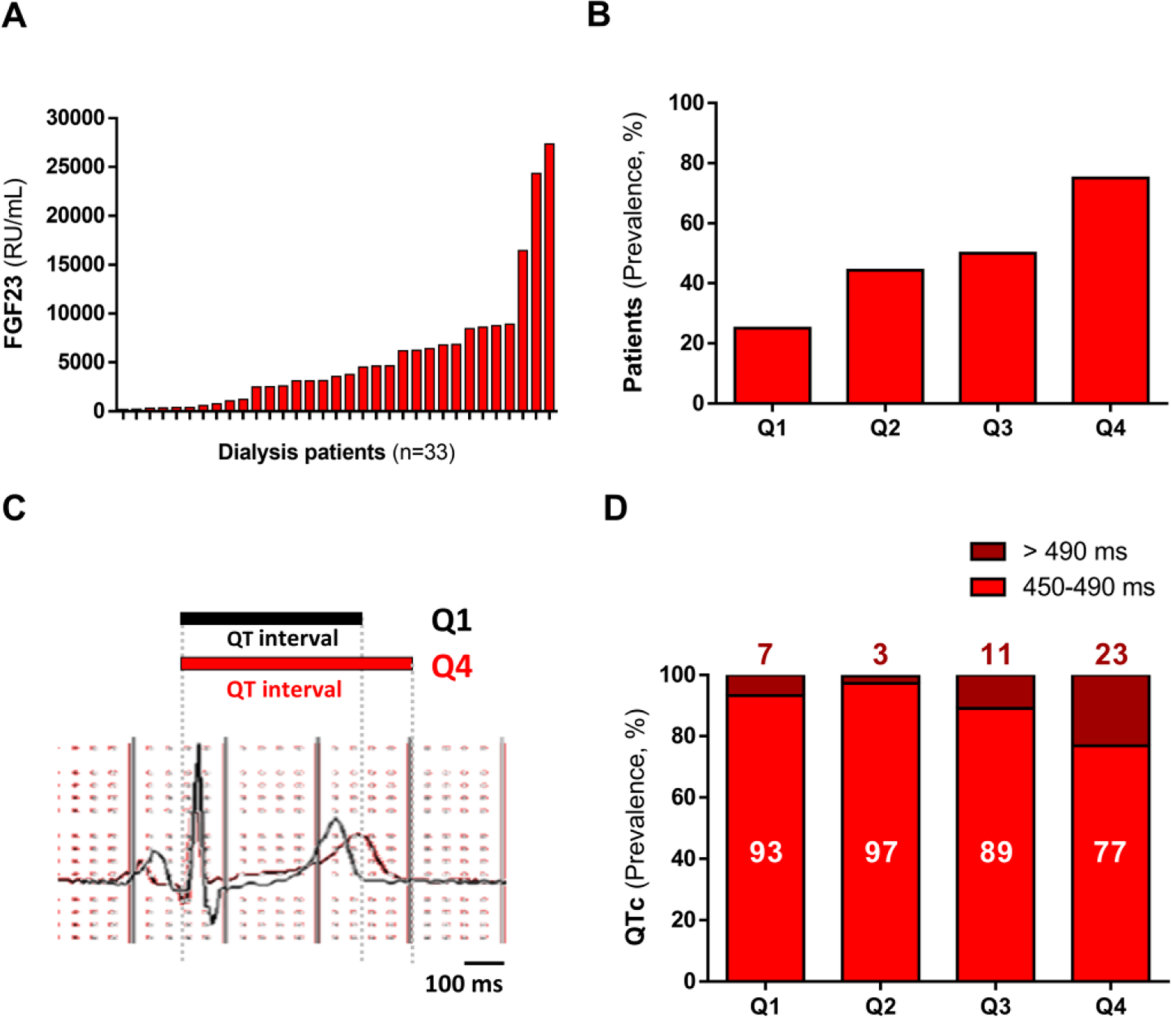
High prevalence of long QT intervals in dialysis-dependent CKD patients with the highest FGF23 levels. **A** Distribution of plasma FGF23 levels in baseline samples of 33 patients with dialysis-dependent CKD. **B** Prevalence of long QT intervals > 450 ms along the FGF23 ascending quartiles. **C** ECG waveforms showing QT interval duration in a representative patient from FGF23 Q1 (black) and from Q4 (red). **D** With increasing quartiles of FGF23, the prevalence of very long (> 490 ms) corrected QT (QTc) intervals significantly increased (*P* < 0.001)

We analyzed the possible relation between serum FGF23 levels and QT interval duration in a cohort of patients with dialysis-dependent CKD. Baseline characteristics of the cohort are shown in Table 1. The mean age of patients was 61 years (51.5% men). Serum FGF23 levels were measured in the cohort (Fig. 1A). The mean level of serum FGF23 in the cohort was 5434 RU/mL, which is 100-fold higher than that described in healthy populations [8]. We split patients into four quartiles according to serum FGF23 levels (Q1–Q4). Age, sex distribution, body mass index (BMI), and systolic and diastolic blood pressure (SBP and DBP, respectively) were similar across the four FGF23 quartiles. Also, no differences were observed in clinical history, including left ventricular hypertrophy (LVH) or previous kidney transplantation (KT). Likewise, serum levels of cholesterol, calcium, phosphorus, 25-hydroxyvitamin D (vitamin D), parathyroid hormone, and potassium (K^+^) were similar across FGF23 quartiles, and no differences were evident in dialysis-related parameters or medications received. All patients underwent 12-lead Holter monitoring from 1 h before the beginning of a dialysis therapy to the start of the next treatment (48 h). When we compared patients according to FGF23 quartile, we found no differences in heart rate (HR) or in HR variability metrics (SDANN, SDNN and MRSSD index) (Table 1). Likewise, no changes were observed for ECG intervals with the exception of QTc interval and T-peak to T-end (TpTe) interval duration, which is an ECG repolarization marker that has been related to increased risk of mortality [40, 41]. QTc intervals > 450 ms were considered prolonged QTc intervals and were called long QTc. We found that the number of patients with long QTc increased as FGF23 levels rose, with a prevalence of 25% of patients in Q1 to 75% in Q4 (Fig. 1B). Two representative ECG traces from Q1 and Q4 patients are shown in Fig. 1C. We performed an in-depth analysis of long QTc by subdividing QTc intervals into moderately long (450–490 ms) and very long QTc (> 490 ms). We found that the probability for very long QTc was higher as FGF23 levels increased (7% in Q1 patients vs. 23% in Q4, *P* < 0.001, Fig. 1D). Furthermore, TpTe significantly increased with higher FGF23 levels (*P* < 0.05, Table 1).

**Table 1:** Demographic data of patients with dialysis-dependent CKD by quartile of circulating FGF23 levels

Fibroblast growth factor 23, RU/mL					
	Q1 [203.2–779.7]	Q2 [1090–3585]	Q3 [3785–6785]	Q4 [6835–27360]	*P*-value
Demographic and clinical characteristics					
Age (years)	64.6 ± 10.6	68.6 ± 14.1	51.3 ± 22.4	59.0 ± 11.8	0.149
Male sex (*n*, %)	6 (75.0)	3 (33.3)	5 (62.5)	3 (37.5)	0.264
BMI (kg/m^2^)	24.3 ± 4.8	21.2 ± 4.4	21.3 ± 2.6	22.2 ± 3.1	0.344
SBP (mmHg)	136.4 ± 26.4	139.0 ± 13.8	122.8 ± 20.7	114.8 ± 23.1	0.088
DBP (mmHg)	78.1 ± 12.0	71.4 ± 18.9	76.5 ± 22.1	67.4 ± 11.4	0.568
Medical history (*n*, %)					
DM	2 (25.0)	1 (11.1)	3 (37.5)	1 (12.5)	0.522
Hypertension	7 (87.5)	8 (88.8)	5 (62.5)	6 (75.0)	0.522
Current smokers	2 (25.0)	0 (0.0)	1 (12.5)	1 (12.5)	0.707
MI	1 (12.5)	1 (11.1)	0 (0.0)	2 (25.0)	0.235
Ischemia	2 (25.0)	0 (0.0)	0 (0.0)	1 (12.5)	0.235
LVH	4 (50.0)	2 (22.2)	5 (62.5)	4 (50.0)	0.387
Previous KT	3 (37.5)	2 (22.2)	3 (37.5)	5 (62.5)	0.403
Laboratory measurements					
Cholesterol (ng/mL)	148.3 ± 38.6	141.7 ± 26.7	147.1 ± 29.0	151.6 ± 25.0	0.922
Calcium (ng/mL)	9.0 ± 0.3	8.8 ± 0.4	9.1 ± 0.5	9.0 ± 0.6	0.747
Phosphorus (ng/mL)	3.9 ± 0.9	4.3 ± 1.2	4.3 ± 1.4	4.9 ± 1.2	0.471
CaxP	35.1 ± 7.8	38.0 ± 10.4	39.3 ± 12.6	43.2 ± 9.5	0.467
25-Hydroxyvitamin D (ng/mL)	9.9 ± 3.1	9.3 ± 3.0	11.2 ± 4.4	8.3 ± 3.0	0.412
Parathyroid hormone (pg/mL)	350.4 ± 138.4	301.4 ± 199.0	475.5 ± 251.2	406.8 ± 242.3	0.444
sKL (ng/mL)	0.4 ± 0.2	0.3 ± 0.1	0.5 ± 0.3	0.3 ± 0.1	0.189
Dialysis-related parameters					
Dialysis vintage (months)	95.4 ± 81.5	58.2 ± 59.3	59.8 ± 50.4	100 ± 100	0.536
Serum creatinine (mg/dL)	6.4 ± 1.3	7.3 ± 2.2	9.1 ± 2.7	7.7 ± 1.9	0.096
Serum albumin (g/dL)	4.3 ± 0.3	3.9 ± 0.3	4.2 ± 0.6	3.9 ± 0.4	0.211
Kt/V	1.7 ± 0.2	1.6 ± 0.3	1.6 ± 0.3	1.6 ± 0.3	0.757
eGFR (ml/min/1.73m^2^)	8.4 ± 2.4	6.5 ± 3.0	6.5 ± 4.2	6.3 ± 2.0	0.447
Medication					
*Anti-hypertensive drugs*					
ACEi/ARB (%)	1 (12.5)	3 (33.3)	4 (50.0)	1 (12.5)	0.260
Beta-blocker (%)	1 (12.5)	5 (55.5)	4 (50.0)	4 (50.0)	0.268
Diuretics (%)	2 (25.0)	0 (0.0)	0 (0.0)	1 (12.5)	0.235
*Anti-arrhythmics drugs*					
Amiodarone (%)	0 (0.0)	0 (0.0)	1 (12.5)	0 (0.0)	0.359
*Bone-mineral metabolism drugs*					
Calcium chelators (%)	1 (12.5)	6 (66.6)	5 (62.5)	5 (62.5)	0.091
Sevelamer (%)	3 (37.5)	4 (44.4)	3 (37.5)	3 (37.5)	0.989
Lantanum carbonate (%)	1 (12.5)	1 (11.1)	5 (62.5)	4 (50.0)	0.054
Cinacalcet (%)	1 (12.5)	3 (33.3)	5 (62.5)	4 (50.0)	0.349
Paricalcitol (%)	7 (87.5)	5 (55.5)	5 (62.5)	4 (50.0)	0.381
Colecalciferol (%)	1 (12.5)	1 (11.1)	0 (0.0)	1 (12.5)	0.785
Parathyroidectomy (%)	0 (0.0)	2 (22.2)	1 (12.5)	1 (12.5)	0.620
Electrocardiogram parameters					
HR (bpm)	78.8 ± 9.3	81.8 ± 13.3	85.8 ± 15.8	83.4 ± 8.3	0.702
SDANN	83.4 ± 30.2	57.6 ± 15.1	76.0 ± 28.0	78.9 ± 15.5	0.153
SDNN	37.3 ± 16.0	33.8 ± 13.5	43.0 ± 13.5	37.5 ± 17.5	0.682
RMSSD	37.8 ± 24.6	45.6 ± 23.1	48.8 ± 13.4	41.5 ± 20.8	0.769
TpTe interval (ms)	85.5 ± 17.3	108.9 ± 39.7	97.1 ± 19.8	136.0 ± 45.6	0.029

Data from 33 patients are reported as mean ± SD or number (percentage). BMI, body mass index; SBP, systolic blood pressure; DBP, diastolic blood pressure, DM, diabetes mellitus; MI, myocardial infarction; eGFR, estimated glomerular filtration rate; LVH, left ventricular hypertrophy; KT, kidney transplantation; eGFR, estimated glomerular filtration rate; HR, heart rate; SDANN, Standard Deviation of the 5 minute Average NN intervals; SDNN, standard deviation of the IBI of normal sinus beats; RMSSD, root mean square of the successive differences; TpTe, T wave peak to T wave end; CaxP, calcium-phosphorus product; sKL: soluble Klotho; ACEi, angiotensin-converting enzyme inhibitor; ARB, angiotensin receptor blocker

### FGF23 prolongs ventricular repolarization and induces long QTc, JT, and TpTe intervals

**Fig. 2:**
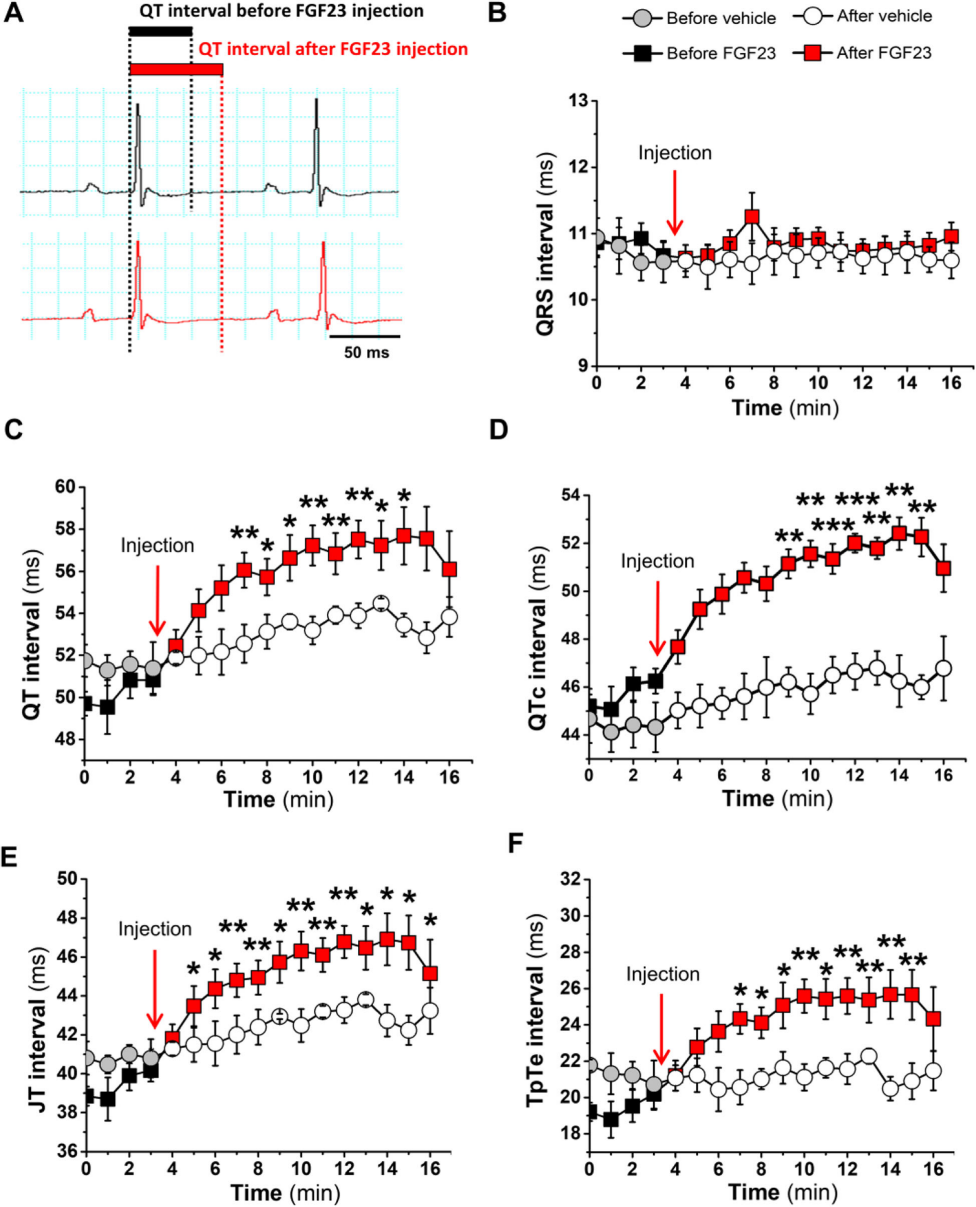
FGF23 induces prolonged ventricular repolarization in healthy mice. **A** ECG waveforms in wild-type healthy mice before (black line) and after (red line) FGF23 administration (40 μg/kg). **B–F** Time course of QRS interval (**B**), QT interval (**C**), QTc interval (**D**), JT interval (**E**), and T peak to T end (TpTe) interval (**F**) measures from ECG recordings after vehicle (*N* = 5 mice) or FGF23 (*N* = 9 mice) administration. Data are presented as mean ± SEM. *P < 0.05, **P < 0.01, and ***P < 0.001 versus baseline

Our data thus far support the concept that FGF23 might be implicated in the prevalence of long QTc in dialysis patients. To test whether FGF23 directly induces in vivo changes in ECG repolarization dynamics, we injected a single intraperitoneal dose of vehicle solution (0.9% sodium chloride) or 40 μg/kg FGF23 in healthy 14-week-old mice and recorded ECGs. We also measured serum FGF23 levels before and after ECG recording. Serum FGF23 levels increased 3.4-fold in mice injected with FGF23, from 231.2 ± 17.0 pg/mL at baseline to 791.5 ± 75.6 pg/mL at the end of ECG recording. Representative ECG traces before (black profile) and after (red profile) FGF23 injection are shown in Fig. 2A. No changes in HR were observed after FGF23 administration (data not shown). No differences were observed in QRS interval (Fig. 2B) after administration, independently of the treatment received. By contrast, QT and QTc intervals were significantly longer in mice treated with FGF23 compared with those receiving vehicle only (Fig. 2C, D). Detailed analysis of the TpTe interval and the JT interval, obtained by subtracting the QRS duration from the QT interval, showed that both were longer in mice receiving FGF23 (Fig. 2E, F).

### Experimental CKD triggers prolonged ventricular repolarization and reduces the repolarizing *I*_tof_

We used 5/6 nephrectomy (Nfx) in mice as an experimental model of chronic renal failure with comparable uremic manifestations to those seen in patients with dialysis-dependent CKD [42]. Macroscopic and biochemical parameters of Sham-operated and Nfx groups are shown in Table 2. No differences were observed in body weight (BW) between groups, and no signs of cardiac hypertrophy were evident in Nfx mice, as indicated by similar heart weight (HW), HW to BW ratio (HW/BW), and cardiomyocyte area in Sham and Nfx mice. While kidney weight (KW) was also comparable between groups, it is important to note that KW in Nfx animals corresponds to the remaining portion of the left kidney (one third) and, accordingly, the left kidney of Nfx mice was hypertrophied (Table 2). Biochemical analysis showed that urea and blood urea nitrogen (BUN) levels were significantly higher in Nfx mice compared with Sham mice (*P* < 0.001, Table 2), whereas no differences in phosphorus levels were apparent, likely as a consequence of the significantly higher levels of FGF23 observed in Nfx mice (*P* < 0.01, Table 2).

**Table 2:** Macroscopic and biochemical parameters in the experimental CKD model and after treatment with recombinant Klotho

	Sham	Nfx	Sham+rKL	Nfx + rKL
Macroscopic parameters				
Body weight (BW, g)	27.7 ± 0.6	25.0 ± 0.8	26.2 ± 0.7	25.3 ± 0.8
Heart weight (HW, mg)	206.7 ± 10.9	178.6 ± 12.4	181.0 ± 4.2	192.4 ± 7.7
HW/BW	7.7 ± 0.2	7.8 ± 0.2	7.2 ± 0.2	7.5 ± 0.3
Cardiomyocyte area (a.u.^2^)	3406 ± 123	3380 ± 146	2958 ± 141	3123 ± 128
Kidney weight (mg)	164.4 ± 0.5	142.7 ± 0.7	153.6 ± 0.3	133.1 ± 0.9
Biochemical parameters				
Urea (mg/dL)	51.7 ± 3.0	112.7 ± 11.1***	42.1 ± 3.9	109.4 ± 11.4^†††^
BUN (mg/dL)	24.2 ± 1.4	52.7 ± 5.2***	19.7 ± 1.8	51.1 ± 5.3^†††^
Phosphorus (mg/dL)	8.3 ± 1.0	7.6 ± 0.8	6.3 ± 0.9	8.2 ± 0.9
FGF23 (pg/mL)	148.9 ± 15.9	345.4 ± 54.2**	123.0 ± 12.0	353.1 ± 49.7^†††^

Data from 8 animals for macroscopic and biochemical parameters per experimental group are reported as mean ± SEM. BW, body weight; HW heart weight; BUN blood urea nitrogen; FGF23, fibroblast growth factor 23. **P < 0.01, ***P < 0.001 vs. Sham; ^†††^*P* < 0.001 vs. Sham+rKL. Sham and Nfx mice were treated with vehicle solution (0.9% sodium chloride)

**Fig. 3:**
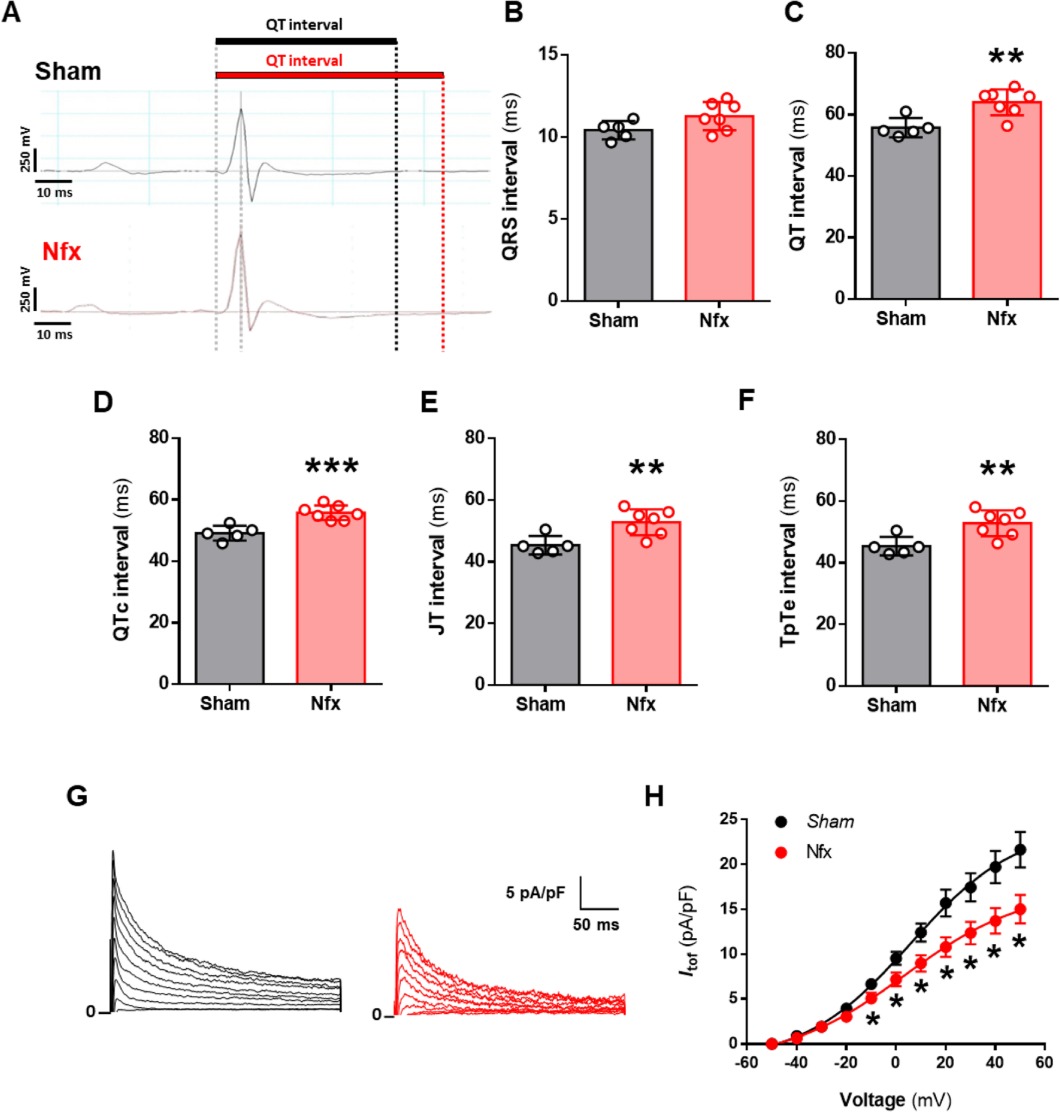
Nfx induces prolonged ventricular repolarization and reduces *I*_*tof*_. **A** ECG waveforms of Sham (black profile, upper panel) and Nfx (red profile, bottom panel) mice. **B–F** Mean values of **(B)** QRS, (**C)** QT, **(D)** QTc, **(E)** JT, and **(F)** TpTe interval duration of Sham (*N* = 5 mice) and Nfx (*N* = 7 mice). **G** Representative *I*_*tof*_ traces recorded from one cardiomyocyte from Sham (left panel, black profile) and Nfx (right panel, red profile) mice. **H** I/V curves for *I*_*tof*_ density obtained in myocytes from Sham (black line, *n* = 24 cells, *N* = 9 mice) and Nfx (red line, *n* = 21 cells, *N* = 7 mice) mice. Data are presented as mean ± SEM. *P < 0.05; **P < 0.01 and ***P < 0.001 vs. Sham

We next analyzed ventricular repolarization dynamics in vivo by ECG. Representative traces from Sham (black profile) and Nfx (red profile) mice are shown in Fig. 3A. No changes were observed in HR (463.6 ± 9.9 ms vs*.* 457.7 ± 13.3 ms for Sham and Nfx, respectively) or in QRS interval (Fig. 3B) between groups. However, Nfx mice displayed significantly prolonged ventricular repolarization characterized by prolonged QT (*P* < 0.01), QTc (*P* < 0.001), JT (*P* < 0.01), and TpTe (both *P* < 0.01) intervals as compared with Sham mice (Fig. 3C–F). Alterations in ventricular repolarization intervals are typically due to changes in the ionic currents involved in ventricular cardiomyocyte repolarization. The most important repolarizing current in the mouse heart is the fast transient outward K^+^ current (*I*_tof_). Representative recording profiles of *I*_tof_ from adult mouse ventricular cardiomyocytes isolated from Sham (black profile, left panel) and Nfx (red profile, right panel) mice are shown in Fig. 3G. Results showed that *I*_tof_ density was significantly lower in Nfx cardiomyocytes than in Sham cardiomyocytes from − 10 to + 50 mV (*P* < 0.05, Fig. 3H).

### Recombinant Klotho treatment on CKD mice prevents both, the prolonged ventricular repolarization, and the reduced *I*_tof_

Because CKD is characterized by Klotho deficiency [43] and Nfx mice have lower Klotho levels than Sham mice (1.2 ng/mL ± 0.2 vs. 11.9 ng/mL ± 5.5, respectively, *P* < 0.001), we chose to treat Nfx mice chronically during 6 weeks with recombinant murine Klotho (rKL). Macroscopic and biochemical parameters are shown in Table 2. No differences were observed for BW, HW, HW/BW ratio, or cardiomyocyte area between Klotho-treated Sham (Sham+rKL) and Klotho-treated Nfx (Nfx + rKL) mice. Furthermore, no differences were observed between vehicle- and rKL-treated mice independently of the surgery. Analogous to the data for vehicle-mice, comparable values for KW were found for both groups, signifying that the left kidney remained hypertrophied. Furthermore, serum urea, BUN, and FGF23 levels were all significantly higher in Nfx+rKL mice than in Sham+rKL mice (*P* < 0.001, Table 2) with no changes to phosphorus levels. Similar values were observed in rKL-treated mice compared to vehicle ones. These data support the notion that chronic Klotho treatment fails to prevent kidney damage.

**Fig. 4:**
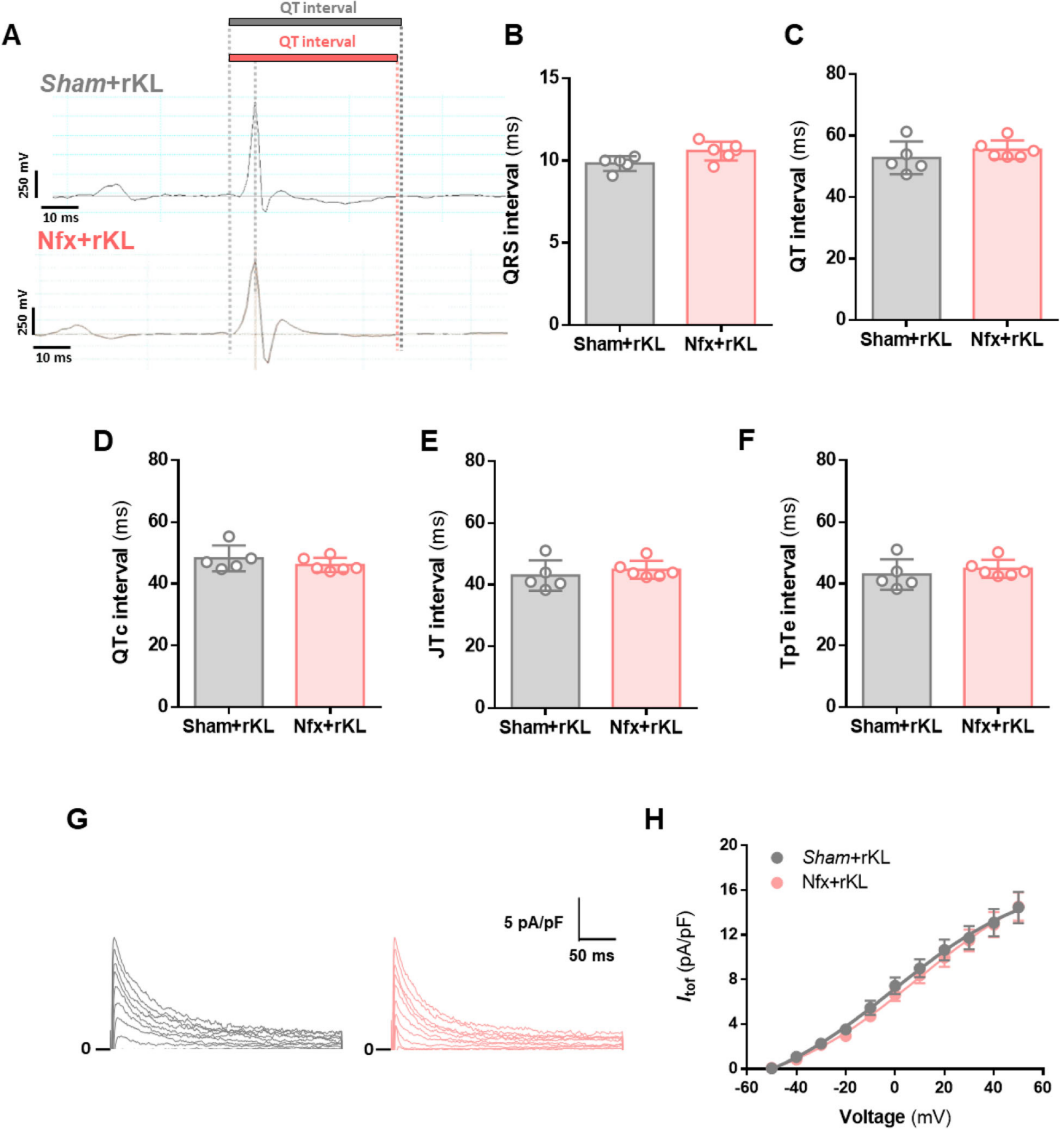
Klotho treatment protects against Nfx-induced prolonged ventricular repolarization and prevents the decreased *I*_tof_ density. **A** ECG waveforms of Sham+rKL (grey profile, upper panel) and Nfx+rKL (pink profile, bottom panel) mice. **B–F** Mean values of **(B)** QRS, **(C)** QT, **(D)** QTc, **(E)** JT, and **(F)** TpTe interval duration of Sham+rKL (*N* = 5 mice) and Nfx + rKL (*N* = 6 mice). **G** *I*_*tof*_ traces recorded in cardiomyocytes from Sham+rKL (left panel, grey profile) and Nfx+rKL (right panel, pink profile). **H** I/V curves for *I*_*tof*_ density obtained in myocytes (bottom panel) from Sham+rKL (grey line, *n* = 23 cells, *N* = 4 mice) and Nfx+rKL (pink line, *n* = 30 cells, *N* = 5 mice) mice. Data are presented as mean ± SEM

We next studied ventricular repolarization dynamics in vivo in Klotho-treated Sham and CKD mice. Representative ECG traces of Sham+rKL (grey profile) and Nfx+rKL (pink profile) mice are shown in Fig. 4A. No changes were observed in QRS interval duration between both groups (Fig. 4B). Recombinant Klotho treatment prevented the changes in ventricular repolarization-related parameters observed in Nfx mice (QT, QTc, JT, and TpTe intervals) (Fig. 4C–F). We then measured *I*_tof_ as before in isolated adult ventricular cardiomyocytes from Klotho-treated mice; representative recording profiles from Sham+rKL (grey line, left panel) and Nfx+rKL (pink profile, right panel) mice are presented in Fig. 4G, showing that *I*_tof_ density was similar in both groups (Fig. 4H).

### Recombinant Klotho treatment counteracts the reduction in *Kv4.2* expression in isolated ventricular cardiomyocytes from CKD mice and in cardiomyocytes exposed to FGF23

**Fig. 5:**
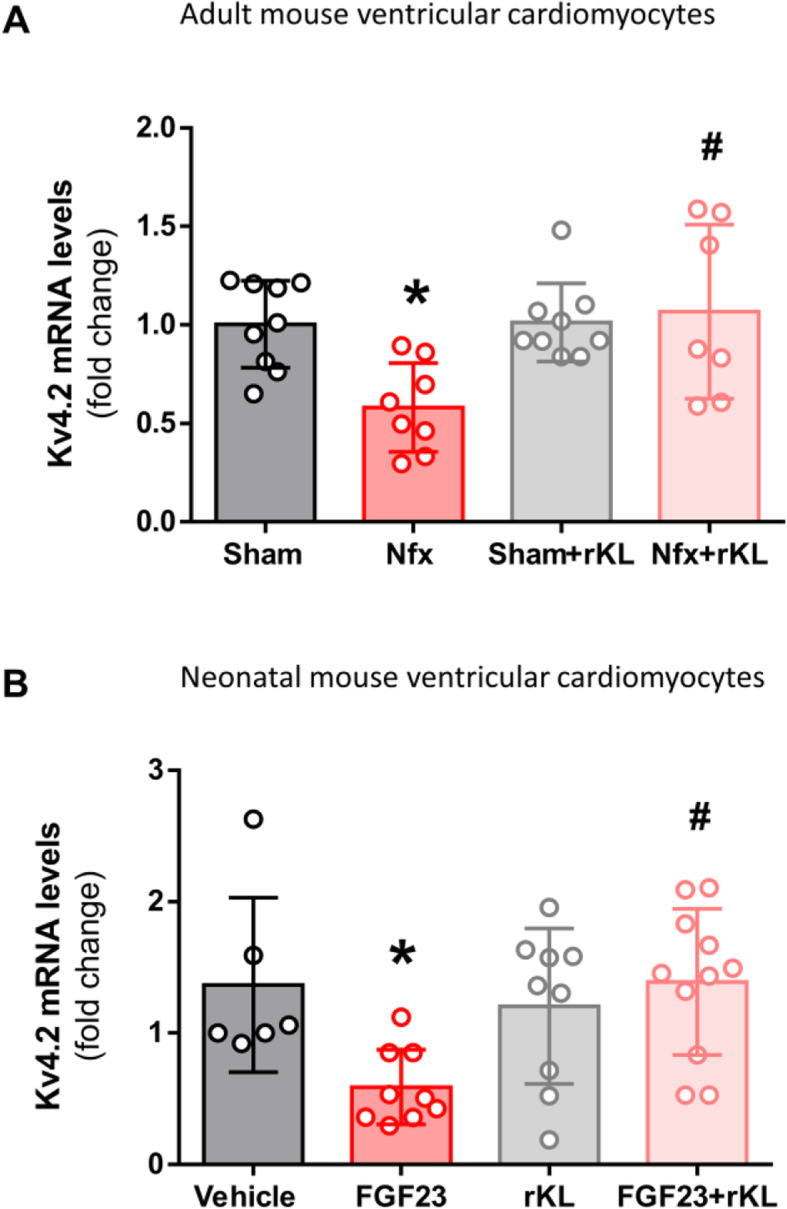
Klotho prevents *Kv4.2* α-subunit downregulation in ventricular myocytes from Nfx and after FGF23 incubation. **A** mRNA expression of *Kv4.2* channel subunit in Sham (*N* = 9 mice), Nfx (*N* = 8 mice), Sham+rKL (*N* = 9 mice), and Nfx+rKL (*N* = 7 mice) mice. **B** mRNA expression of *Kv4.2* in neonatal ventricular cardiomyocytes incubated with vehicle (*N* = 6 replicate wells), FGF23 (*N* = 9 replicate wells), rKL (*N* = 9 replicate wells), and FGF23 + rKL (*N* = 11 replicate wells). Data are presented as mean ± SEM. *P < 0.05 vs. the corresponding control (Sham or vehicle-neonatal cardiomyocytes) and ^#^*P* < 0.05 vs. FGF23-neonatal cardiomyocytes

As the reduced density of *I*_tof_ in Nfx mice could be due to changes in the levels of the K^+^ channels, we analyzed the mRNA expression of the main α-subunit that encodes the *I*_*tof*_ channel in mice, *Kv4.2*. *Kv4.2* expression was significantly lower in ventricular myocytes from Nfx mice than from Sham mice (*P* < 0.05, Fig. 5A). By contrast, analysis of *Kv4.2* expression in isolated adult ventricular myocytes from *Sham*+rKL and Nfx+rKL mice revealed no significant difference in levels between both groups (Fig. 5A). However, *Kv4.2* expression was found significantly higher in Nfx+rKL compared to Nfx group (*P* < 0.05, Fig. 5A).

The mineral bone component FGF23 is a uremic factor known to be directly involved in cardiac (including cardiomyocyte) damage [44, 45]. We therefore studied the direct effect of FGF23 on K^+^ channel expression in healthy cardiomyocytes. Because the prolonged culture of adult ventricular cardiomyocytes results in poor survival and loss of phenotype [46], we used neonatal mouse ventricular myocytes to analyze the effect of FGF23 on *Kv4.2* expression. The results showed that *Kv4.2* expression was significantly lower in neonatal mouse cardiomyocytes exposed to 100 ng/mL FGF23 for 48 h than to vehicle solution (*P* < 0.05, Fig. 5B), suggesting that FGF23 represses *Kv4.2* channel subunit expression. By contrast, neonatal cardiomyocytes pre-incubated with recombinant Klotho prior to exposure with FGF23 did not show a decrease in *Kv4.2* subunit expression (Fig. 5B), and expression was significantly higher than in cells incubated only with FGF23 (*P* < 0.05, Fig. 5B).

### Klotho overexpression prevents prolonged ventricular repolarization in CKD mice, and transgenic mice with reduced Klotho expression exhibit prolonged ventricular repolarization

**Fig. 6:**
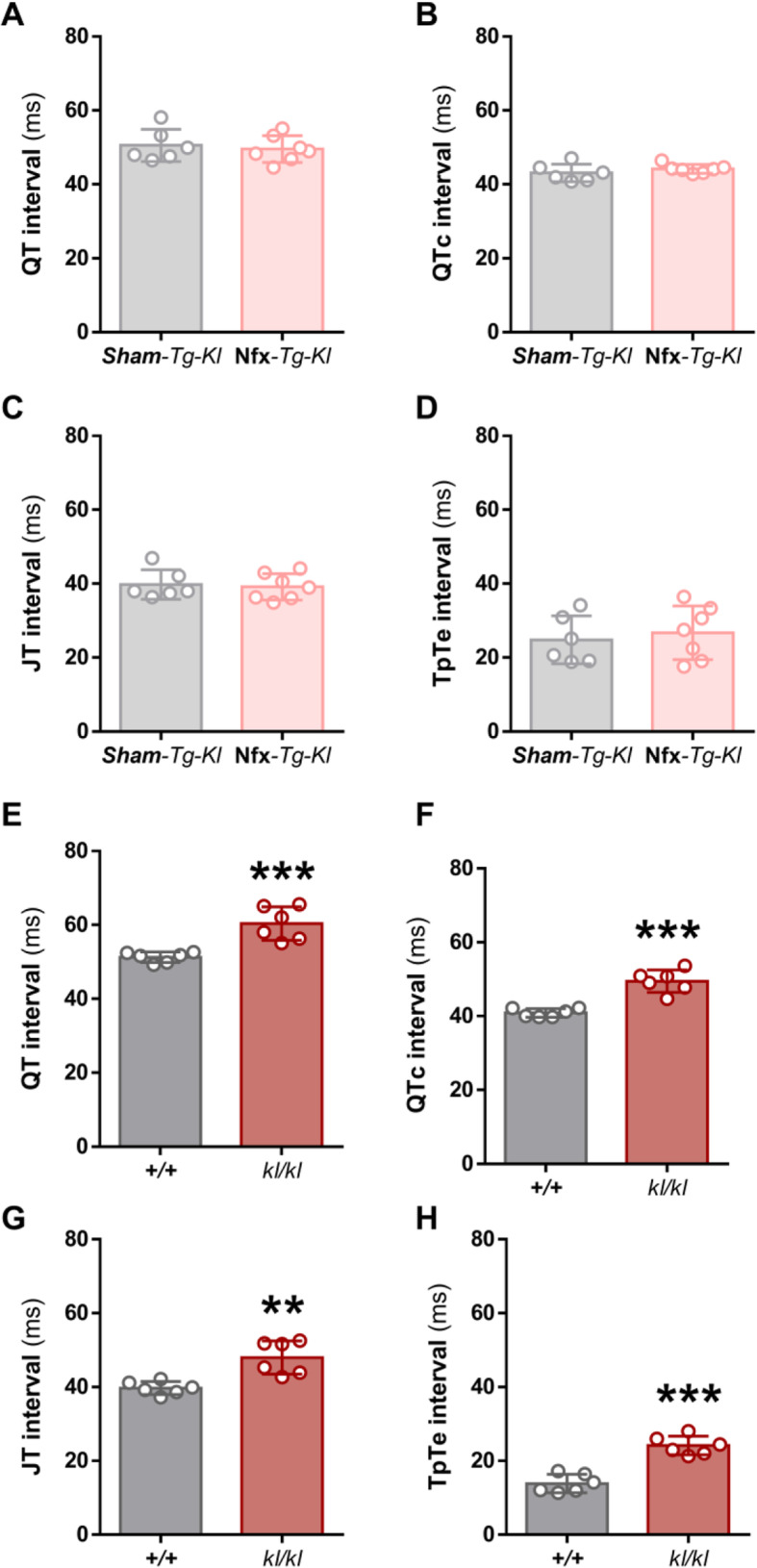
Klotho overexpression prevents prolonged ventricular repolarization induced by Nfx and Klotho deficiency prolongs ventricular repolarization. **A–D** Mean values of **A** QT, **B** QTc, **C** JT, and **D** TpTe interval duration of *Sham-Tg-kl* (*N* = 6 mice), Nfx-*Tg-kl* (*N* = 7 mice). **E–H** Mean values of **(E)** QT, (**F)** QTc, **(G)** JT, and **(H)** TpTe intervals duration of +/+ (*N* = 6 mice), *kl*/*kl* (*N* = 6 mice). Data are presented as mean ± SEM. **P < 0.01 and ***P < 0.001 vs. +/+

Given the above findings, we next sought to analyze the ventricular repolarization-related parameters in a transgenic mouse model characterized by enhanced endogenous Klotho expression. Klotho-overexpressing mice (*Tg-Kl*) underwent Nfx surgery to investigate whether high endogenous Klotho levels would prevent the prolongation of the QT interval induced by CKD. Macroscopic and biochemical parameters of Sham*-Tg-Kl* and Nfx-*Tg-Kl* groups are shown in Additional File 1. No differences were observed in BW, HW, and HW/BW ratio between the two groups and, as before, similar values of KW were found between both groups indicating that the left kidney remained hypertrophied. Biochemical analysis revealed significantly higher levels of urea, BUN, and FGF23 in Nfx-*Tg-Kl* mice than in Sham*-Tg-Kl* mice (*P* < 0.01, Additional File 1) with no evident differences in phosphate levels. Analysis of ECG recordings from Sham*-Tg-Kl* and Nfx-*Tg-Kl* mice revealed no differences in QT, QTc, JT, or TpTe intervals (Fig. 6A–D), indicating that Klotho overexpression protects against acquired prolonged ventricular repolarization in Nfx mice.

Finally, to elucidate the contribution of Klotho to the prolongation of ventricular repolarization beyond renal dysfunction, we examined ECG parameters in mice hypomorphic for Klotho (*kl/kl*), which show very high systemic levels of FGF23, and in wild-type (*+/+*) littermates. Macroscopic and biochemical parameters of the two groups are shown in Additional File 2. Klotho hypomorphic mice were smaller than their wild-type littermates, with lower BW and HW (both *P* < 0.001, Additional File 2) but a higher HW/BW ratio (*P* < 0.05, Additional File 2). *Kl/kl* mice had impaired renal function with higher urea, BUN and FGF23 levels than *+/+* mice (*P* < 0.01, *P* < 0.01, and *P* < 0.001, respectively, Additional File 2). No changes were observed in phosphate levels between both groups, which might be due to the phosphaturic effect of the extremely high FGF23 found in *kl/kl* mice. Analysis of ventricular repolarization *in vivo* revealed a significant increase in QT and QTc in *kl/kl* mice (*P* < 0.001, Fig. 6E, F). Furthermore, *kl/kl* mice exhibited prolonged JT (*P* < 0.01, Fig. 6G) and TpTe (*P* < 0.001, Fig. 6H) intervals with respect to *+/+* mice.

## Discussion

**Fig. 7:**
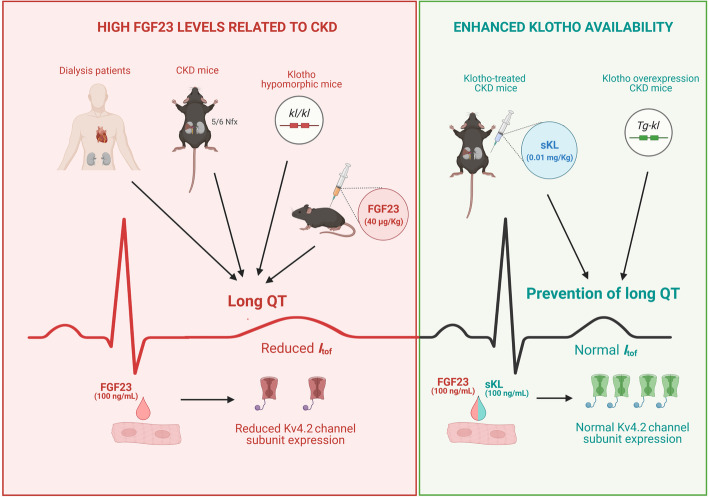
Diagram representing the involvement of FGF23-Klotho axis in acquired long QT syndrome. CKD, chronic kidney disease; FGF23, fibroblast growth factor 23; *I*_*tof*_, fast transient outward potassium current; *kl/kl*, Klotho hypomorphic mice; Kv4.2, K^+^ channel 4.2 subunit; Nfx, nephrectomy; sKL, soluble Klotho; Tg-kl, Klotho overexpression mice

CKD induces premature aging [47], which is frequently associated with reduced expression of the anti-aging factor Klotho [43, 48]. Aging has been shown to increase the prevalence of a wide range of pathologies including cardiovascular diseases [49], which are the main cause of death in the elderly [50], particularly in patients with CKD [51]. In this line, fatal arrhythmias and SCD are the leading sources of mortality in patients with CKD, especially in dialysis-dependent patients [52, 53]. This latter group frequently has asymptomatic arrhythmias [54] and alterations in ventricular repolarization with significant QT interval prolongation [20]. Here, we describe for the first time a relevant association between high FGF23 levels and the presence of long QT in patients with dialysis-dependent CKD, and we show that enhancing Klotho availability protects against acquired long QT in uremic milieu a in a mouse model of CKD (Fig. 7).

Klotho-deficient mice are characterized by premature aging and reduced lifespan [11]. We found that mice with reduced Klotho expression showed prolonged QTc, TpTe, and JT intervals. Interestingly, loss of Klotho expression in mice is linked to the development of cardiac hypertrophy [55], cardiac dysfunction [36], and a pro-arrhythmic phenotype [35], although Klotho is not expressed in the heart under basal conditions [11]. In the present study, we show that enhancing Klotho availability through exogenous Klotho supplementation or endogenous Klotho overexpression prevented the prolonged ventricular repolarization in an experimental CKD model, pointing to Klotho administration as a potential therapeutic strategy to preserve ventricular rhythm. In the clinical context, reduced levels of Klotho have been related to an increased risk for cardiovascular diseases [56], supporting its proposal as a predictor of all-cause mortality in the elderly [57]. Indeed, no association has been found between Klotho levels and cardiovascular mortality in the general population [58], indicating that Klotho is likely not a good biomarker of cardiovascular risk. This is in contrast to the MBD component FGF23, whose role as a cardiovascular risk biomarker is better established [59]. We found that CKD mice and Klotho hypomorphic mice in particular present high FGF23 serum levels. In the latter case, the very high systemic levels of FGF23 that accompany the defect in Klotho expression might be the primary responsible factor for the cardiac alterations related to the prolonged ventricular repolarization observed in CKD.

Most previously published reports on the cardiac effects of FGF23 have focused on its structural role based on hypertrophy development [44, 60, 61] and on rhythm alterations almost restricted only to atrial fibrillation [62–64]. For example, treatment of the atrial cell line HL-1 with FGF23 triggers pro-arrhythmogenic events [65]. Also, it was recently demonstrated that FGF23 induces a pro-arrhythmogenic phenotype in ventricular adult cardiomyocytes by altering intracellular Ca^2+^ handling [66, 67]. We establish here that FGF23 has a direct effect on ventricular repolarization in vivo. Translating these results to a clinical setting, we show for the first time to our knowledge that FGF23 levels are associated with QT interval prolongation in a cohort of patients with dialysis-dependent CKD. Prolonged QT interval appears to be common in dialysis patients [24, 68] and has been related to increased risk of SCD [20, 23]. Prolonged QT interval is typically a consequence of cardiac structural changes [69], although some studies have described prolonged QT interval in the absence of cardiac hypertrophy [70]. Supporting this latter idea, our analysis showed no differences in the presence of left ventricular hypertrophy across FGF23 quartiles. These results together with the absence of cardiac hypertrophy in the CKD mouse model indicate that the alterations in cardiac repolarization are associated to the high FGF23 and low Klotho levels present in uremia in the absence of relevant structural cardiac remodeling, although it is important to note that our CKD model presents with other uremia-associated alterations, and, therefore, it is not possible to claim that FGF23 is the sole responsible factor for the increased QT interval in the context of CKD. That being said, intraperitoneal administration of FGF23 in healthy mice demonstrated that it can directly induce QT interval prolongation. A recent study has demonstrated that this FGF23-induced prolongation of the QT interval in healthy mice was mediated by FGFR4 [71]. However, in our experimental model of CKD, we did not find any evidence of cardiac hypertrophy development, which is usually associated to FGFR4-mediated FGF23 signaling. Moreover, no changes were detected in FGFR4 expression in hearts of Nfx mice (data not shown). These experimental data together with the clinical data in dialysis patients where the prevalence of LVH along FGF23 quartiles were similar support the possibility that FGF23 requires other mechanisms to induce QT prolongation. Also, no association has yet been reported between the QT interval and cardiac structure in dialysis patients [72]. The relationship between FGF23 and the development of LVH in CKD patients has been described [44], but contradictory data has been reported in dialysis patients. Some studies described an association between FGF23 and LVH in dialysis patients [73] while others authors did not find associations between serum FGF23 levels and LVH [74, 75]. Thus, the QT interval prolongation observed in our cohort might indeed be due to the increased levels of FGF23 in the dialysis patients, which is also experimentally supported by the increased QT interval duration observed in healthy mice after a single FGF23 injection.

The QT interval is not the only ECG parameter that has been associated with SCD. The TpTe interval has also been used in the measurement of the transmural dispersion of ventricular repolarization [76] and is also related to increased risk of mortality [40, 41]. We found that the TpTe interval in dialysis patients was significantly longer in Q4 FGF23 than in Q1 FGF23. A prolonged TpTe interval has been proposed as a predictor of ventricular arrhythmias and SCD [41, 77]. Moreover, in patients with HF, TpTe has been found to be longer in those patients with CKD [78]. Supporting these data is the finding of a significant reduction in TpTe interval after renal transplantation [79], demonstrating a reduction in the risk of developing cardiac arrhythmia after transplantation compared with those patients with end-stage renal failure. Adverse electrophysiological remodeling such as abnormal K^+^ currents is one of the mechanisms related to prolonged ventricular repolarization. Indeed, loss-of-function mutations involving the K^+^ current channel have been described as the cause of long QT syndrome [80, 81], and reduced *I*_*tof*_ has been related to long QT syndrome in rodents [82, 83]. In mice, *I*_*tof*_ is one of the most important current in determining ventricular repolarization duration due to the extraordinarily abbreviated action potentials [84]. Our study shows that Nfx model exhibits decreased *I*_*tof*_ density, and a significant reduction of this K^+^ current has been found in a less aggressive CKD model in rats [85]. Moreover, similar results have been found in an established animal model of HF where a reduction in cardiomyocyte K^+^ current was linked to prolonged QT, TpTe, and JT intervals [39]. Downregulated K^+^ currents might be caused by changes in K^+^ channel expression [86] in which Kv4.2 is the main channel responsible for maintaining ventricular repolarization in mice [87]. Kv4.2 downregulation has been found by some authors in failing hearts [88] and has been related to the development of early arrhythmias in mice [89], including long QT phenotype [83]. We observed a significant reduction of *Kv4.2* expression in adult cardiomyocytes from Nfx mice, and we found that treatment of neonatal ventricular cardiomyocytes with FGF23 also downregulated *Kv4.2* expression. These findings mechanistically define a role for FGF23 in ventricular repolarization prolongation observed in experimental and clinical uremia.

The prolongation of QT, TpTe, and JT intervals observed in CKD mice are also present in the transgenic mice model of reduced Klotho expression that is accompanied by a marked increase in FGF23 levels. These findings point to the need for therapeutic strategies to prevent/block the effects of FGF23 on the heart in the context of uremia. Along this line, the importance of the balance between FGF23 levels and Klotho availability is gaining attraction, and several studies have demonstrated a relevant cardioprotective role of soluble Klotho in the context of cardiac hypertrophy by direct regulation of ion channels [90] or even in the context of uremic cardiomyopathy [13, 35, 36]. Moreover, it has been recently described that FGF23-induced cardiac hypertrophy in mice is attenuated by soluble Klotho administration [91]. The present work demonstrates that improving Klotho availability prevents the acquired long QT syndrome associated with CKD. Klotho-treated Nfx mice did not develop prolonged repolarization-related intervals, likely due to the prevention of reduced *I*_tof_. Similarly, increased endogenous Klotho availability through Klotho overexpression protected against repolarization abnormalities in Nfx mice. Finally, the downregulation in *Kv4.2* mediated by FGF23 exposure was also blocked in cardiomyocytes pre-treated with recombinant Klotho. We believe that these findings are clinically relevant as they point to Klotho not only as a mechanism linked to cardiorenal damage but also as a useful therapeutic target to prevent fatal arrhythmias triggered by FGF23 in CKD.

### Limitation

A key question that remains to be solved is how the cardioprotective actions of Klotho treatment against FGF23 effect occur and whether this protection depends on the blockade of the FGF23 binding to FGFRs. In our hands, we did not observe any evidence of cardiac hypertrophy neither changes in the expression of FGFRs in the heart of CKD mice (data not shown). These results support the possibility that rKL could induce a switch in the signaling of FGF23 from FGFR4 to FGFR1 under high Klotho levels, which has been recently proposed by other authors [91, 92]. Further studies will be needed to elucidate the mechanisms underlying Klotho cardioprotection.

## Conclusions

Our results support FGF23 as a promoting factor of cardiac arrhythmias related to prolonged QT interval. In this context, enhancing Klotho availability appears to be a valid therapeutic strategy to prevent acquired long QT syndrome and potentially fatal ventricular arrhythmias and SCD in CKD.

## Supplementary Information

**Additional file 1. **Macroscopic and biochemical parameters in *Tg-kl* mice.

**Additional file 2.** Macroscopic and biochemical parameters in Klotho hypomorphic mice.

## Data Availability

The datasets generating during and/or analyzed during the current study are available from the corresponding authors on reasonable request.

## Abbreviations

ACEi: Angiotensin-converting enzyme inhibitor
ARB: Angiotensin receptor blocker
BMI: Body mass index
BUN: Blood urea nitrogen
BW: Body weight
CaxP: Calcium-phosphorus product
CKD: Chronic kidney disease
cQT: Corrected QT interval
DBP: Diastolic blood pressure
ECG: Electrocardiogram
eGFR: Estimated glomerular filtration rate
FGF23: Fibroblast growth factor 23
FGFR: Fibroblast growth factor receptor
HF: Heart failure
HW: Heart weight
HR: Heart rhythm
*I*_*tof*_: Fast transient outward potassium current
KT: Kidney transplantation
KW: Kidney weight
LVH: Left ventricular hypertrophy
MBD: Mineral bone disorders
Nfx: Nephroctomy
rKL: Recombinant Klotho
RMSSD: Root mean square of the successive differences
SBP: Systolic blood pressure
SCD: Sudden cardiac death
SDANN: Standard Deviation of the 5 minute Average NN intervals
SDNN: Standard deviation of the IBI of normal sinus beats
*Tg-kl*: Transgenic Klotho overexpressing mice
TpTe: T wave peak to T wave end
