# Supplementary material for: The anti-aging factor Klotho protects against acquired long QT syndrome induced by uremia and promoted by fibroblast growth factor 23
Source: BMC Med. 2022 Jan 19;20:14. doi: 10.1186/s12916-021-02209-9 (PMC8767669; doi:10.1186/s12916-021-02209-9)
Supplement: Supplementary file 1 — Additional file 1. Macroscopic and biochemical parameters in Tg-kl mice. [file 12916_2021_2209_MOESM1_ESM.docx]

**Additional file 1. Macroscopic and biochemical parameters in *Tg-kl* mice.**

|  | Sham-*Tg-kl* | Nfx- *Tg-kl* |
| --- | --- | --- |
| Macroscopic parameters | | |
| Body weight (BW, g) | 25.1 ± 1.2 | 23.2 ± 1.0 |
| Heart weight (HW, mg) | 194.2 ± 4.4 | 201.8 ± 6.4 |
| HW/BW | 7.8 ± 0.3 | 8.7 ± 0.3 |
| Kidney weight (mg) | 157.8 ± 9.1 | 136.8 ± 13.9 |
| Biochemical parameters | | |
| Urea (mg/dL) | 53.4 ± 4.5 | 130.1 ± 18.2****** |
| BUN (mg/dL) | 24.9 ± 2.1 | 60.8 ± 8.5****** |
| Phosphorus (mg/dL) | 10.4 ± 1.1 | 8.9 ± 0.5 |
| FGF23 (pg/mL) | 108.3 ± 39.9 | 551.6 ± 70.0****** |

Data from 5 animals for macroscopic and biochemical parameters per experimental group are reported as mean ± SEM. BW: Body weight; HW: heart weight; BUN: blood urea nitrogen; FGF23: fibroblast growth factor 23. ^**^*P* < 0.01 vs. Sham-*Tg-kl*.
