# Supplementary material for: The anti-aging factor Klotho protects against acquired long QT syndrome induced by uremia and promoted by fibroblast growth factor 23
Source: BMC Med. 2022 Jan 19;20:14. doi: 10.1186/s12916-021-02209-9 (PMC8767669; doi:10.1186/s12916-021-02209-9)
Supplement: Supplementary file 2 — Additional file 2. Macroscopic and biochemical parameters in Klotho hypomorphic mice. [file 12916_2021_2209_MOESM2_ESM.docx]

**Additional file 2. Macroscopic and biochemical parameters in Klotho hypomorphic mice.**

|  | +/+ | *kl/kl* |
| --- | --- | --- |
| Macroscopic parameters | | |
| Body weight (BW, g) | 24.6 ± 0.3 | 8.3 ± 0.6******* |
| Heart weight (HW, mg) | 186.3 ± 11.3 | 93.0 ± 10.9******* |
| HW/BW | 7.5 ± 0.4 | 11.5 ± 1.6***** |
| Kidney weight (mg) | 167.7 ± 8.6 | 55.8 ± 4.2******* |
| Biochemical parameters | | |
| Urea (mg/dL) | 34.9 ± 2.6 | 62.9 ± 7.8****** |
| BUN (mg/dL) | 16.3 ± 1.2 | 29.4 ± 3.7****** |
| Phosphorus (mg/dL) | 17.3 ± 0.9 | 19.2 ± 1.3 |
| FGF23 (pg/mL) | 239.0 ± 29.9 | 363837 ± 43152******* |

Data from 6 animals for macroscopic and biochemical parameters per experimental group are reported as mean ± SEM. BW: Body weight; HW: heart weight; BUN: blood urea nitrogen; FGF23: fibroblast growth factor 23. ^*^*P* < 0.05, ^**^*P* < 0.01 and ^***^*P* < 0.001 vs. +/+.
